# Multiple Furuncular Cutaneous Myiasis in a 4‐Month‐Old Infant in an Urban Setting: A Case Report From Cameroon

**DOI:** 10.1155/crpe/9016315

**Published:** 2026-05-28

**Authors:** Isabelle Mekone Nkwele, Géraldine Lynn Ewane Ngole, Christelle Nadia Mpah Edimo, David Chelo

**Affiliations:** ^1^ Department of Pediatrics, Faculty of Medicine and Biomedical Sciences, University of Yaoundé I, Yaoundé, Cameroon, uy1.uninet.cm

**Keywords:** Cameroon, *Cordylobia anthropophaga*, cutaneous myiasis, infant

## Abstract

Furuncular cutaneous myiasis is a parasitic infestation of the skin by dipteran larvae, commonly reported in tropical Africa, and underreported in infants. We report the case of a 4‐month‐old male infant from Yaoundé, Cameroon, who presented with incessant crying and a rapidly progressive skin eruption over 4 days. Examination revealed multiple inflammatory nodules at varying stages of development; serous fluid was observed exuding from a central punctum, with intermittent larval movement. Environmental history identified risk factors, including poor household hygiene, drying clothes in shaded areas or indoors, infrequent ironing, and high fly density. The diagnosis was established clinically and confirmed by manual extraction of larvae, consistent with *Cordylobia anthropophaga* based on clinical and epidemiological features, although no entomological confirmation was performed. Treatment consisted of gentle extraction, local antiseptic baths, and topical antibiotic ointment. Clinical evolution was favorable, with rapid resolution of symptoms following extraction, no emergence of new lesions, and complete healing within 3 months, leaving mild postinflammatory hyperpigmentation and small atrophic scars. This case is remarkable for the high burden of lesions, occurrence in an urban environment, and the young age of the patient. It highlights the importance of considering myiasis in the differential diagnosis of nodular eruptions in infants in endemic areas and emphasizes preventive measures such as proper drying and ironing of clothes and reducing fly exposure.

## 1. Introduction

Furuncular cutaneous myiasis is a tissue infestation caused by larvae of certain flies, encountered mainly in tropical regions with high fly density and poor hygiene. In Sub‐Saharan Africa, the most frequently implicated species is *Cordylobia anthropophaga* (commonly known as the Tumbu fly or Cayor worm), whose females lay eggs on damp clothes or soil [1, 2]. Upon contact with the skin, body temperature stimulates larval hatching and penetration [3].

Although widely described in rural endemic areas, furuncular cutaneous myiasis remains underreported in infants, particularly in urban areas. We report a case of multiple furuncular cutaneous myiasis in a 4‐month‐old infant living in Yaoundé, Cameroon.

## 2. Case Presentation

A four‐month‐old male infant, born at term with a birth weight of 4250 g, with no significant past medical history and no evidence of immunodeficiency, presented for evaluation due to persistent crying and an evolving skin eruption over a 4‐day period.

**FIGURE 1:**
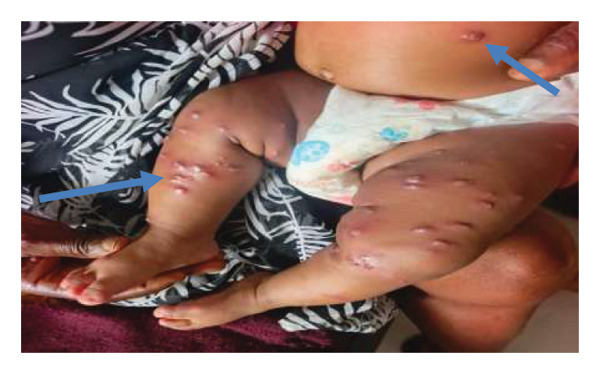
Skin lesions at presentation after 4 days of evolution in a 4‐month‐old infant: multiple erythematous inflammatory nodules, in different stages of development, variably centered by a punctum (arrows).

The illness began as a progressive maculopapular erythematous eruption involving both lower limbs, nonpruritic, with surrounding areas of unaffected skin and lesions in different stages of development. Over 4 days, the lesions increased in size, becoming nodular with a central punctum (figure 1). The lesions discharged serous fluid, and some showed live larval movement. The eruption subsequently extended to the trunk and upper limbs, becoming generalized while sparing the face.

The environmental assessment identified risk factors, including drying clothes indoors, infrequent ironing, and high household fly density. Immunizations were up to date, and the feeding history revealed exclusive breastfeeding until 3 months, followed by early weaning and the introduction of infant formula.

On admission, the infant was irritable without fever or signs of dehydration or systemic involvement. Physical examination showed multiple inflammatory nodules of varying stages on the trunk and limbs. Each lesion was centered by a punctiform orifice, sometimes enlarged, oozing serous fluid, and in some lesions, larval mobility was visible. The nodules were painful on palpation, explaining the persistent crying. No significant lymphadenopathy or septic signs were observed. No formal entomological identification of the larvae was performed. The diagnosis of *Cordylobia anthropophaga* was based on the clinical presentation and local epidemiological context.

**FIGURE 2:**
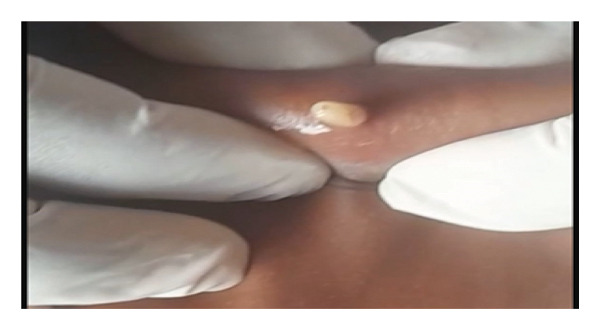
Extraction of larvae by manual expression.

**FIGURE 3:**
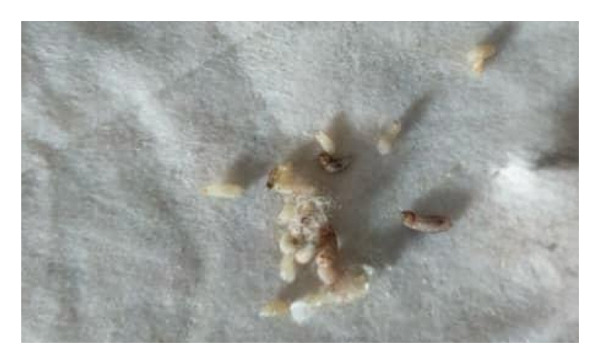
Larvae of *Cordylobia anthropophaga* extracted from cutaneous lesions.

Management consisted of gentle and progressive manual extraction (Figure 2). No occlusive substance was used prior to extraction. Manual expression was preferred due to the visibility of the larval posterior end and the accessibility of the lesions, allowing successful removal without rupture. More than 40 larvae were extracted (Figure 3). Local care included regular antiseptic baths.

A topical antibiotic ointment was applied; however, its benefit in the absence of secondary bacterial infection is uncertain, and it was unlikely to have significantly influenced the outcome.

No systemic antibiotics were administered, as there were no signs of bacterial superinfection.

**FIGURE 4:**
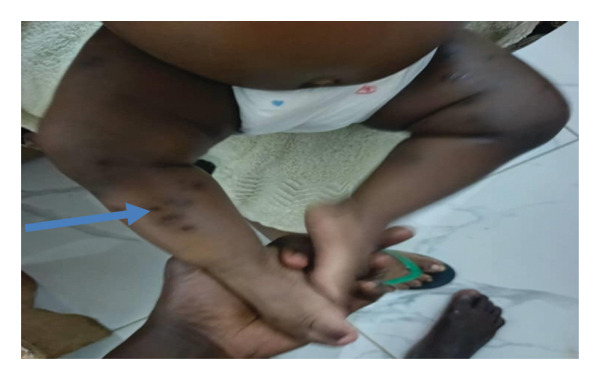
Evolution in 3 months following treatment: resolution of active lesions, with residual postinflammatory hyperpigmentation and small atrophic scars (arrow).

The clinical outcome was favorable, with complete resolution at 3 months. Following extraction, the infant showed rapid clinical improvement, with a marked reduction in pain and irritability within 48 h. No new lesions appeared after treatment. The nodular lesions resolved progressively over the following weeks, leaving minor sequelae including postinflammatory hyperpigmentation and small atrophic scars (Figure 4).

Preventive measures were equally implemented to improve domestic hygiene, such as sun‐drying and ironing of clothes, and to reduce household fly burden.

## 3. Discussion

Furuncular cutaneous myiasis, although common in tropical Africa, is underreported in infants and in urban areas. In this case, the absence of recent travel to rural areas in our patient suggests that the infestation was likely acquired in an urban environment, indicating that transmission can occur within cities under favorable hygienic conditions. Established risk factors including drying clothes in shaded or indoor environments, absence of ironing, and high fly density were clearly present in this case [2, 4]. This urban occurrence may reflect the persistence of environmental and hygienic conditions favorable to fly breeding within cities, including inadequate waste management and household practices such as drying clothes in shaded areas.

In a recent African review, *Cordylobia anthropophaga* accounted for about two‐thirds (66.2%) of documented cases [1].

Clinically, furuncular nodules centered by a central breathing pore, sometimes allowing visualization of the larvae, are highly suggestive of cutaneous myiasis. Diagnosis is mainly clinical and confirmed by larval extraction. Several extraction techniques have been described, including occlusive methods to induce larval emergence by occluding the breathing pore. Common agents include petroleum jelly, adhesive tapes/films, commercial bacon strips, tobacco paste, and paraffin. In our case, manual extraction was sufficient due to the superficial location of the larvae and their visible posterior ends. The diagnosis was based entirely on clinical and epidemiological features, representing a limitation of the report. We suspected *Cordylobia anthropophaga*, the most commonly implicated species in Sub‐Saharan Africa. We do not consider other species, such as *Dermatobia hominis*, which is primarily found in the tropical and temperate zones of Central and South America.

Differential diagnoses, including skin infections such as impetigo or bacterial abscesses, may potentially lead to misdiagnosis in the absence of visible larvae [5].

The main complications of furuncular lesions include local tenderness and bacterial superinfection, which in rare cases may progress to systemic infection.

The management is based on mechanical extraction of larvae, combined with local antiseptic care. Prognosis is favorable if larvae are completely removed and preventive measures are implemented, such as sun‐drying and ironing of clothes and reduction of household fly density [4, 6].

Furuncular cutaneous myiasis is a self‐limiting condition, given that larvae eventually complete their development and spontaneously exit the skin. Treatment primarily aims to reduce tenderness, shorten disease duration, and prevent complications such as bacterial superinfection and scarring.

This case is notable for the burden of lesions and unusual infection, in a young infant, within an urban setting. Similar cases reported in infants are scarce, with most described in rural areas [7]. Our observation in an urban environment underscores that the risk is not limited to traditional endemic zones and supports the need for clinical vigilance, even in cities.

## 4. Conclusion

Furuncular cutaneous myiasis in infants is underreported; it should be promptly recognized when inflammatory nodules with central puncta are encountered, particularly in tropical settings with poor hygiene. Early treatment with extraction and local care ensures favorable outcomes. Prevention is based on improved and the reduction of household fly exposure.

## Author Contributions

Dr. Isabelle Mekone Nkwele conceptualized and collected the data and drafted the manuscript. Dr Géraldine Lynn Ewane Ngole critically revised the manuscript. Dr Christelle Nadia Mpah Edimo critically revised the manuscript. Pr. David Chelo revised and approved the final version to submit.

## Funding

The authors declare that there is no funding associated with the publication of this article.

## Disclosure

The authors approved the final manuscript as submitted and agreed to be accountable for all aspects of the work.

## Ethics Statement

This case report was conducted in accordance with institutional and international ethical standards. Written informed consent was obtained from the patient’s parents prior to inclusion, after provision of an information sheet. All data were collected in compliance with confidentiality and anonymity standards, and no identifiable information is included in this report.

## Consent

Written informed consent was obtained from the patient’s parents for publication of this case report and accompanying images.

## Conflicts of Interest

The authors declare no conflicts of interest.

## Data Availability

Data sharing is not applicable to this article as no datasets were generated or analyzed during the current study.
